# Antioxidant role of melatonin against nicotine’s teratogenic effects on embryonic bone development

**DOI:** 10.22038/IJBMS.2018.26705.6539

**Published:** 2018-08

**Authors:** Halil Yılmaz, Tolga Ertekin, Emre Atay, Mehtap Nisari, Hatice Susar Güler, Özge Al, Ahmet Payas, Seher Yılmaz

**Affiliations:** 1 Department of Therapy and Rehabilitation, Kozakli Vocational School, Nevsehir Haci Bektas Veli University, Nevsehir, Turkey; 2 Department of Anatomy, Faculty of Medicine, Afyonkarahisar Health Sciences University, Afyonkarahisar, Turkey; 3 Department of Anatomy, Faculty of Medicine, Erciyes University, Kayseri, Turkey; 4 Department of Anatomy, Faculty of Medicine, Bozok University, Yozgat, Turkey; 5 Department of Therapy and Rehabilitation, Sungurlu Vocational School, Hitit University, Corum, Turkey

**Keywords:** Bone, Melatonin, Nicotine, Ossification, Rat

## Abstract

**Objective(s):**

This study investigated the possible effects of low (3 mg/kg) and high (6 mg/kg) doses of nicotine on the skeletal development of rat fetuses by the double staining method and the protective role of melatonin (10 mg/kg) against these effects.

**Materials and Methods:**

Eighteen adult female Wistar-Albino rats were divided into six groups (n=3, each) as control, low-dose nicotine, high-dose nicotine, low-dose nicotine+melatonin, high-dose nicotine + melatonin and melatonin. While nicotine was given to the experimental groups on gestation days 1–20, nicotine and melatonin were administered together to the treatment groups. The fetuses were delivered by cesarean section on the 20^th^ day of pregnancy. The skeletal systems of the fetuses were stained using the double staining method. The forelimbs and hindlimbs of the fetuses were firstly investigated under a stereomicroscope, and then their photos were taken. The total bone length, the length of the ossified part and the ossification rate were calculated using the ImageJ program.

**Results:**

The degree of ossification in the bones of the feet and the hands was determined. When the total bone length and the length of the ossified part were evaluated, they were significantly decreased in the nicotine groups (*P<*0.05), but were close to each other in the treatment and the control groups (*P<*0.05).

**Conclusion:**

It has been found that the use of nicotine during pregnancy delays skeletal ossification and that melatonin, a powerful antioxidant, eliminates the teratogenic effects of nicotine.

## Introduction

Skeletal development is an important part of somatic growth and development (1). Bone tissue is an active tissue that is sensitive to hormonal, metabolic and nutritional conditions (2-5). However, pregnant women may be exposed to some chemicals due to their living conditions. These chemicals can have several teratogenic effects on the embryo, depending on their characteristics (6, 7).

Smoking is an addiction with harmful consequences for human health due to containing hazardous chemicals. Nicotine is the main addictive chemical in tobacco**.** It comprises about 95% of the total alkaloid content and approximately 0.5–8.0% of the dry weight of tobacco. Other alkaloids are nornicotine, anabasine, cotinine, and nicotine-N-oxide (8). A single cigarette usually contains 0.6-2 mg of nicotine (9). Nicotine is a compound that can cross the placenta and enter into the fetal circulation (10).

Active cigarette smoking or passive exposure to cigarette smoke during pregnancy causes fetal defects (11). It has been reported in the literature that the use of nicotine during pregnancy delays fetal bone development and increases the number of partially ossified bones (12). Several studies have reported that the effects of nicotine on bone development depend on the exposed dose, it reduces osteoblast activity and increases osteoclast activity (13), and it delays wound healing by reducing the regeneration of fibroblasts and macrophages (14). It has also been reported that it reduces the storage of vitamin D, which plays an important role in bone development, in the body (15, 16). Recent studies have reported that nicotine causes damage by disrupting the oxidant-antioxidant balance and that antioxidants are useful against these damages (11, 17).

Melatonin, also known as N-acetyl-5-methoxy tryptamine, is secreted by the epiphyseal gland (pineal gland), especially at night (18). Melatonin, which plays a role in tasks such as biological rhythm regulation, cell renewal, strengthening the immune system and body temperature regulation, is a powerful antioxidant and free radical scavenger (19, 20). 

In the literature, there are very few studies examining the teratogenic effects of nicotine on fetal bone development. However, there was no study investigating the protective effects of melatonin against these negative effects on fetal bone development. The aim of this study is to investigate the bone damage due to smoking different doses of nicotine during pregnancy and the protective effects of melatonin against nicotine using the double staining method. 

## Materials and Methods

***Animal selection***

For the study, the Local Ethics Committee of Erciyes University Animal Experiments was applied and Ethics Committee decision number of 15/97 dated on 12.08.2015 was taken. Rat selection was made according to the literature (3, 5, 9, 10). For this purpose, 18 adult female (5-7 months old) Wistar-Albino rats weighing 180-220 g, which were used in this study, were obtained from the Erciyes University Experimental Research and Application Center. Two female rats and one male rat were placed in the cages at 05.00 pm for mating. Vaginal smears were taken from the female rats at 07.00 am the next morning and were examined under a microscope. The female rats that had a sperm-positive vaginal smear were accepted to be on day 0.5 of pregnancy. They were kept at constant temperatures of 19-21 ^º^C and on a 12 hrs of light-12 hrs of darkness in specially prepared, automatically air-conditioned rooms. They were fed with normal pellet diet.

***Experimental groups***

The pregnant rats were randomly divided into 6 groups (n=3). Doses (5, 8-11) and the injection (5, 8, 10) route were determined according to the literature. According to the literature in double skeletal staining studies (1, 3, 5, 6), injections were made every day on gestation days 1–20. 

Control group: Normal saline was administered intraperioteanally (IP).

Low-dose nicotine group: 3 mg/kg nicotine was administered by subcutaneous (SC) injection.

Low-dose nicotine+melatonin group: 3 mg/kg nicotine was administered by SC injection and 10 mg/kg melatonin were administered IP.

High-dose nicotine group: 6 mg/kg nicotine was administered by SC injection.

High-dose nicotine+melatonin group: 6 mg/kg nicotine was administered by SC injection and 10 mg/kg melatonin were administered IP.

Melatonin group: 10 mg/kg melatonin was administered IP.

Nicotine was administered at two doses in the morning (07:00) and evening (16:30) in the high-dose nicotine group and at single dose in the evening (16:30) in the low-dose nicotine group. Melatonin was administered half hour after nicotine administration (17:00), taking circadian rhythm into consideration.

***Preparation of injections***

Nicotine and melatonin powders 98% were obtained from Sigma-Aldrich. Normal saline was used as a solvent solution to adjust the amount of nicotine to be given to the rats. To provide pH balance, phosphate-buffered saline (PBS) were used to dissolve melatonin powder. Both materials were prepared daily and stock solution was not made.

***Manipulation of rats and obtaining fetuses***

The pregnant rats were anesthetized with ketamine (75 mg/kg)+xylazine (10 mg/kg) on the 20^th^ day of pregnancy. The abdominal area of the rats was cleaned with 70% ethyl alcohol, and then the anterior abdominal wall was removed with a transverse incision. The uteruses and the fetuses were dissected together with the placentas. The fetuses were first examined macroscopically. The fetal weight was estimated, and the fetal crown–rump length was measured with digital calipers. 

***Double skeletal staining in fetuses***

In order to stain the skeletal system of the fetuses by the double staining method, all fetuses were kept in 70% ethyl alcohol for 4-7 days, and thus the water was allowed to withdraw. Following this procedure, they were kept in pure acetone for 1-3 days to clear their oil. Then, their skins were peeled, and their internal organs and eyes were removed. The fetuses were moved from acetone into a double staining solution formed with Alizarin Red-S (100 mg) and Alcian Blue (300 mg) in glass containers. They were incubated for 7 days in an oven whose temperature was set at 38-40 ^o^C, and thus the tissues were allowed to be stained. At the end of the 7^th^ day, the fetuses were washed under running tap water for 2 hours to enter the reaction with water. Subsequently, it was passed to the transparency stage with 1% potassium hydroxide (KOH). The stained fetuses were then kept in 20%, 50%, 80% and finally 100% pure glycerin.

For morphometric measurements, the fetus extremities were photographed on a stereomicroscope by the Nikon E5700 digital camera. Then, the obtained photographs were transferred to the computer. Length and area measurements of the bone to be examined in the transferred photographs were performed using the ImageJ program (http://rsb.info.nih.gov/ij/docs/index.htlm). The ossification rate in long bones was calculated using the data from area measurements.

***Statistical analysis***

The total bone length, the length of the ossified part, the total bone surface area and the ossification surface area in bones treated with double skeletal staining were measured with the ImageJ program. The obtained data were analyzed using the IBM SPSS (Statistical Package for the Social Sciences) Statistics 22 program, which is compatible with the Windows 64-bit operating system. One-way ANOVA test was performed after the original data was entered into Excel. After determining the normal distribution, tukey test was applied in a 95% confidence interval with *post-hoc* multiple comparison command. The Pearson chi-square test was performed to analyze the degree of ossification in the bones of the feet and the hands. According to the analysis result, *P*<0.05 was considered statistically significant.

## Results

***Effects on growth parameters***

The crown–rump length and weight of the fetuses were shown that when 3 mg/kg and 6 mg/kg nicotine were given, there was a statistically significant decrease in these parameters (*P*<0.05). When 10 mg/kg melatonin was administered as a protective agent, it was determined that growth parameters were significantly increased close to the control group.

***Effects on the metacarpal bones***

**Figure 1:**
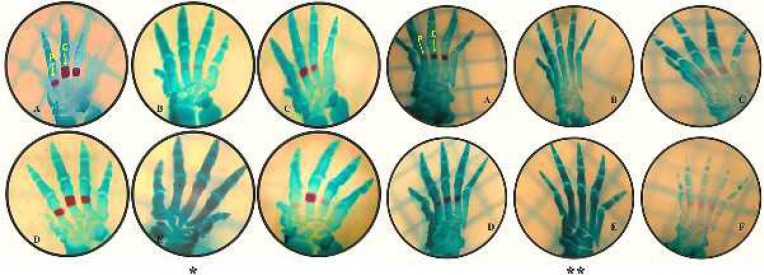
Images of the bones of the hands and feet. (A) Control group. (B) Low-dose nicotine group. (C) Low-dose nicotine+melatonin group. (D) Melatonin group. (E) High-dose nicotine group. (F) High-dose nicotine+melatonin group. (C; complete ossification, P; partial ossification, * hands, ** feet)

When we investigated 20-day-old rat fetuses, it was observed that only the metacarpal bones were ossified. 150 metacarpal bones were examined in each group. While 76 bones were completely ossified in the control group, 30 bones were completely ossified in the low-dose nicotine group. Complete ossification was not observed in any of the bones in the high-dose nicotine group (*P*<0.05). When nicotine and melatonin were administered together, the degree of ossification and the number of ossified bones increased (*P*<0.05) (Figure 1) (Table 1).

***Effects on the upper extremity long bones***

**Figure 2:**
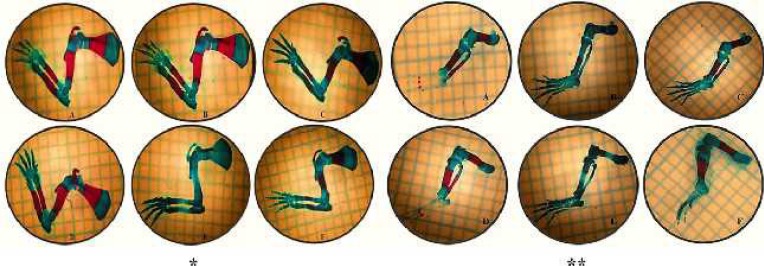
Images of the upper and lower extremity bones. (A) Control group. (B) Low-dose nicotine group. (C) Low-dose nicotine + melatonin group. (D) Melatonin group. (E) High-dose nicotine group. (F) High-dose nicotine + melatonin group. (* Upper Extremity Bones, ** Lower Extremity Bones)

In our study, upper extremity long bones (humerus, radius, and ulna) were evaluated. In general, when 3 mg/kg nicotine was given, it was determined that there was a statistically significant decrease in the length of the ossified part and the ossification rate in these bones (*P*<0.05). Ossification was found to be significantly lower in the 6 mg/kg nicotine group than in the 3 mg/kg nicotine group (*P*<0.05). When nicotine and melatonin were administered together, ossification increased and was close to the control group (Figure 2) (Table 2).

***Effects on the metatarsal bones***

When we looked at 20-day-old rat fetuses, it was observed that only the metatarsal bones were ossified. The degree of ossification of the metatarsal bones was determined. 150 metatarsal bones were examined in each group. In the control group, 90 metatarsal bones were completely ossified, 30 metatarsal bones were partially ossified, and 30 metatarsal bones were completely cartilaginous. When we evaluated the number of completely ossified bones, 30 metatarsal bones were completely ossified and 76 metatarsal bones were partially ossified in the low-dose nicotine group, while in the high-dose nicotine group, complete ossification was not observed in any of the bones, 24 metatarsal bones were partially ossified and 126 metatarsal bones were completely cartilaginous. When melatonin was given as a protective agent, 82 metatarsal bones were completely ossified and 38 metatarsal bones were partially ossified in the low-dose nicotine+melatonin group, while 62 metatarsal bones were completely ossified and 28 metatarsal bones were partially ossified in the high-dose nicotine + melatonin group. When nicotine was given, ossification in the metatarsal bones decreased (*P*<0.05). When nicotine and melatonin were administered together, it was determined that ossification in the metatarsal bones was significantly increased and was close to the control group (*P*<0.05) (Figure 1), (Table 3).

***Effects on the lower extremity long bones***

The lower extremity long bones (femur, tibia, and fibula) were evaluated in our study. In general, when 3 mg/kg nicotine was given, it was determined that there was a statistically significant decrease in the length of the ossified part and the ossification rate in these bones. When the nicotine dose was increased up to 6 mg/kg, it

was found that this decrease was significantly increased (*P*<0.05). When nicotine and melatonin were administered together (low-dose nicotine+melatonin, high-dose nicotine + melatonin), it was determined that ossification was significantly increased and was close to the control group (*P*<0.05) (Figure 2) (Table 4). 

## Discussion

Smoking is an addiction with harmful consequences for human health (21). Nicotine, which is an addictive substance found in tobacco smoke, can pass through the placenta by 88% efficiency and may enter into the fetal circulation (22). In studies investigating the teratogenic effects of nicotine during pregnancy, nicotine has been used at different doses ranging from 1 mg/kg to 9 mg/kg (23-28). In general, most of studies preferred 3 mg/kg nicotine and 6 mg/kg nicotine as low and high nicotine doses, respectively (25-29). As a result, nicotine was administered to rats via subcutaneous injections at doses of 3 and 6 mg/kg in our study. 

**Table 1:** Ossification rate of the metacarpal bones

	**Number of data (n)**	**Complete ossification **	**Partial ossification**	**No ossification **
**Control**	150	76	14	60
**Low-dose nicotine **	150	30^a^,^c^,^e^	54	66
**Low-dose nicotine + melatonin**	150	66	24	60
**High-dose nicotine**	150	0^a^,^b^,^d^,^e^	18	132
**High-dose nicotine +melatonin**	150	58	32	60
**Melatonin**	150	78	12	60

Chi-square test; *P<*0.05 was considered statistically significant;

(a) It is significant when compared with the control group;

(b) It is significant when compared with the low-dose nicotine+melatonin group;

(c) It is significant when compared with the high-dose nicotine+melatonin group;

(d) It is significant when compared with the melatonin group;

(e) It is significant when compared with the low-dose nicotine group

**Table 2:** Ossification rate of the upper extremity long bones

**Group**	**N**	**Humerus**			**Ulna**			**Radius**		
		**Total bone length**	**Length of ossified part**	**Ossification rate** **(%)**	**Total bone length**	**Length of ossified part**	**Ossification rate (%)**	**Total bone length**	**Length of ossified part**	**Ossification rate (%)**
**Control**	30	4.19±0.04	1.93±0.20	43.71±2.33	4.12±0.08	1.94±0.13	46.08±2.82	3.24±0.10	1.54±0.12	40.80±3.73
**LDN**	30	4.11±0.08	1.68±0.18^a^,^b^,^d^	38.79±5.74^a^,^b^,^d^	4.06±0.15	1.62±0.21^a^,^b^,^d^	35.77±4.72^a^,^b^,^d^	3.18±0.10	1.24±0.14^a^,^b^,^d^	35.54±5.68^a^,^b^,^d^
**LDN+ MEL**	30	4.17±0.19	1.94±0.19	42.93±3.98	4.12±0.28	1.89±0.17	42.86±6.33	3.20±0.21	1.47±0.18	39.58±5.95
**HDN**	30	3.87±0.14^a^,^c^,^d^,^e^	1.28±0.17^a^,^c^,^d^,^e^	31.42±5.33^a^,^c^,^d^,^e^	3.74±0.19^a^,^c^,^d^,^e^	1.23±0.30^a^,^c^,^d^,^e^	30.32±5.14^a^,^c^,^d^,^e^	2.93±0.25^a^,^c^,^d^,^e^	1.05±0.15^a^,^c^,^d^,^e^	31.36±5.98,^c^,^d^,^e^
**HDN+ MEL**	30	4.14±0.09	1.81±0.09	40.83±3.06	4.07±0.14	1.83±0.13	42.14±4.82	3.16±0.16	1.37±0.09^a^,^d^	40.02±4.73
**MEL**	30	4.20±0.11	1.89±0.11	42.94±2.35	4.09±0.15	1.89±0.15	47.60±4.83	3.21±0.15	1.50±0.13	39.44±4.54

ANOVA test; *P<*0.05 was considered statistically significant; LDN: low-dose nicotine; HDN: high-dose nicotine; MEL: melatonin;

(a) It is significant when compared with the control group;

(b) It is significant when compared with the LDN+MEL group;

(c) It is significant when compared with the HDN+MEL group;

(d) It is significant when compared with the MEL group;

(e) It is significant when compared with the LDN group

**Table 3:** Ossification rate of the bones of the feet

**Group**	**Number of data (n)**	**Complete ossification **	**Partial ossification **	**No ossification **
**Control**	150	90	30	30
**LDN**	150	30^a^,^c^,^e^	76	44
**LDN + MEL**	150	82	38	60
**HDN**	150	0^a^,^b^,^d^,^e^	24	126
**HDN + MEL**	150	62	28	60
**MEL**	150	90	30	60

Chi-square test, *P<*0.05 was considered statistically significant; LDN: low-dose nicotine; HDN: high-dose nicotine; MEL: melatonin;

(a) It is significant when compared with the control group;

(b) It is significant when compared with the LDN+MEL group;

(c) It is significant when compared with the HDN+MEL group;

(d) It is significant when compared with the MEL group;

(e) It is significant when compared with the LDN group

**Table 4:** Ossification rate of the lower extremity long bones

**Group**	**N**	**Femur**			**Tibia**			**Fibula**		
		**Total bone length**	**Length of ossified part**	**Ossification rate (%)**	**Total bone length**	**Length of ossified part**	**Ossification rate (%)**	**Total bone length**	**Length of ossified part**	**Ossification rate (%)**
**Control**	30	3.85±0.21	1.28±0.10	31.08±3.14	3.79±0.14	1.42±0.13	35.06±4.88	3.45±0.09	1.39±0.14	38.80±4.88
**LDN**	30	3.66±0.16	0.91±0.15^a^,^b^,^d^,^e^	23.10±3.17^a^,^b^,^d^,^e^	3.63±0.17	1.04±0.14^a^,^b^,^d^	26.04±4.96^a^,^b^,^d^	3.44±0.15	0.94±0.10^a^,^b^,^d^,^e^	24.16±5.53^a^,^b^,^d^,^e^
**LDN + MEL**	30	3.87±0.29	1.34±0,16	29.76±4.41	3.78±0.32	1.40±0.23	34.85±4.62	3.42±0.29	1.29±0.35	38.91±4.08
**HDN**	30	3.51±0.19^a^,^c^,^d^,^e^	0.78±0.15^a^,^c^,^d^,^e^	19.34±3.94^a^,^c^,^d^,^e^	3.25±0.23^a^,^c^,^d^	0.84±0.37^a^,^c^,^d^	23.40±5.55^a^,^c^,^d^	3.01±0.19^a^,^c^,^d^,^e^	0.76±0.19^a^,^c^,^d^,^e^	20.92±4.89^a^,^c^,^d^,^e^
**HDN + MEL**	30	3.76±0.18	1.19±0.11	26.99±4.00	3.50±0.18	1.27±0.11	34.47±3.62	3.23±0.19	1.25±0.17	29.26±4.18
**MEL**	30	3.88±0.15	1.27±0.11	30.79±2.72	3.85±0.18	1.42±0.13	34.49±2.42	3.41±0.15	1.32±0.12	38.34±4.19

ANOVA test; *P<*0.05 was considered statistically significant; LDN: low-dose nicotine; HDN: high-dose nicotine; MEL: melatonin;

(a) It is significant when compared with the control group;

(b) It is significant when compared with the LDN+MEL group;

(c) It is significant when compared with the HDN + MEL group;

(d) It is significant when compared with the MEL group;

(e) It is significant when compared with the LDN group

In this study, it was found that the crown–rump length was significantly decreased in the nicotine groups compared to the control group. Birth weight was measured as 2.40 g in the control group, 2.20 g in the low-dose nicotine group and 1.99 g in the high-dose nicotine group. There was a statistically significant difference between the groups in terms of birth weight (*P*<0.05). All of the demographic findings in our study are consistent with the literature (30-32). 

Double skeletal staining is a reliable and objective method that is used for a long time in teratogenic studies (1, 33-35).

In the study in which Ozturk *et al.* examined the development of anterior limb bones in healthy rat fetuses by the double staining method, they found that the mean bone length on the 20^th^ day of pregnancy was 5.4±.16 mm in the humerus, 5.2±0.2 mm in the ulna and 4±0.16 mm in the radius (36). In our study, it was determined that the mean bone length in 20-day-old rat fetuses in the control group was 4.19±0.04 mm in the humerus, 4.12±0.08 mm in the ulna and 3.24±0.1 mm in the radius. The reason of the numerical differences between the two studies is the use of different methods to estimate gestational age. Although Öztürk *et al.* reported that sperm-positive vaginal smear was considered as day 0 of pregnancy, we designated the sperm-positive vaginal smear as day 0.5 of pregnancy. 

It has been reported in the literature that the effects of nicotine on bone development depend on the dose of exposure and that high doses of nicotine cause bone tissue destruction by reducing osteoblast activity and increasing osteoclast activity (13). In the study in which Kurtoglu *et al.* investigated the adverse effects of nicotine on bone development, nicotine (3 mg/kg IP) was given to maternal rats during pregnancy and lactation. They reported at the end of the experiment that the birth weights of 21-day-old rat offspring receiving nicotine were 5.47±0.39 g that were significantly lower than that of the control group, and the femur lengths of 21-day-old rat offspring receiving nicotine were significantly lower than that of the control group (control: 21.1±0.3 mm, experimental group: 19.1±1.6 mm) (25). 

Carmines *et al*. investigated the teratogenic effect of nicotine in female rat fetuses exposed to cigarette smoke before pregnancy (2 weeks) and during pregnancy (20 days). It was reported that 150 mg TPM/m^3^ (11.2 ± 1.09 mg/m^3 ^nicotine) did not show teratogenic effect, but there was a significant increase in the number of missing or non-ossified bones (supraoccipital, sternebrae) in the groups exposed to 300 mg TPM/m^3^ (18.6 ± 2.07 mg/m^3 ^nicotine) and 600 mg TPM/m^3^ (41.8 ± 3.71 mg/m^3 ^nicotine) (12). Similar to the study of Kurtoglu *et al.,* we found that the total bone length, the length of the ossified part and the ossification rate of long bones in the anterior and posterior extremities in the fetuses of nicotine-treated mothers were significantly lower than that in the control group. When the degree of ossification in the bones of the feet and the hands was examined in our study, the number of the metacarpal and metatarsal bones showing complete ossification was less in the low-dose nicotine group compared to the control group. Moreover, complete ossification was not observed in any of the bones of the feet and the hands in the high-dose nicotine group (25). The negative relationship between the degree of ossification and nicotine dose in our study was similar to the findings of Carmines *et al.* (12).

Several studies have shown that nicotine has teratogenic effect by disrupting the oxidant-antioxidant balance in living organisms (17, 37-39). No significant effects on embryofetal development were observed in rats given melatonin (40, 41). In recent years, the protective effect of natural or synthetic antioxidants against nicotine has attracted the attention of researchers, and one of the most powerful of these antioxidants is melatonin. It has been suggested that the administration of 10 mg/kg melatonin in maternal rats has a protective effect against the teratogenic effects of nicotine on various organs in fetal development (28, 42, 43). In our study, we determined the protective effect of melatonin at 10 mg/kg similar to the literature. 

Baykan *et al.* investigated the role of melatonin in preventing nicotine-induced myocardial injury in the offspring of pregnant rats treated with nicotine during pregnancy and lactation. In addition, melatonin (10 mg/kg) was given to the Sprague-Dawley rats in the high-dose (6 mg/kg) and low-dose (1 mg/kg) for 11 weeks from the start of pregnancy to the end of lactation. They reported that melatonin (10 mg/kg) reduced the nitric oxide (NO) and myocardial and plasma malondialdehyde (MDA) levels (free radicals) and increased the plasma glutathione peroxidase (GSHPx) and superoxide dismutase (SOD) levels, and thus showed a protective effect against myocardial damage caused by nicotine (28).

## Conclusion

When the long bones (humerus, radius, ulna, femur, tibia, fibula) in the upper and lower extremities were examined in our study, it was concluded that the administration of melatonin against the teratogenic effect of nicotine on bone development in experimental groups increased ossification rate by 10% in the 3 mg/kg nicotine group and increased ossification rate by 22% in the 6 mg/kg nicotine group. The findings obtained with melatonin administration are consistent with other literature. It was determined that the administration of 10 mg/kg melatonin plays a protective role against the teratogenic damage of nicotine on the skeletal system. 

According to our findings, in proportion to nicotine dose in the fetuses of pregnant rats exposed to nicotine, the number of low-birth-weight rat offspring increased, their crown-rump length was reduced, the number of completely ossified bones decreased, bone development was delayed, and thus ossification rate decreased. It was determined that when melatonin was given against nicotine, their birth weight and height increased close to the control group, the number of non-ossified bones decreased, and normal bone development was achieved. We think that melatonin has a strong protective role against nicotine in bone development due to its antioxidant properties, and that our results will benefit future studies of melatonin.
